# Tickborne Encephalitis Virus, Northeastern Italy

**DOI:** 10.3201/eid1210.060395

**Published:** 2006-10

**Authors:** Anna Beltrame, Maurizio Ruscio, Barbara Cruciatti, Angela Londero, Vito Di Piazza, Roberto Copetti, Valentino Moretti, Paolo Rossi, Gian Luigi Gigli, Luigia Scudeller, Pierluigi Viale

**Affiliations:** * University of Udine, Udine, Italy;; † Hospital of San Daniele, San Daniele, Italy;; ‡ Hospital of Pordenone, Pordenone, Italy;; § San Antonio Abate Hospital, Tolmezzo, Italy;; ¶ Hospital of Udine, Udine, Italy

**Keywords:** TBEV, Epidemiology, Italy, letter

**To the Editor:** Approximately 3,000 cases of tickborne encephalitis virus (TBEV) disease are registered annually in Europe (*1*). In Italy, indigenous TBEV infection cases have been only sporadically recorded from 1975 through 2001; in addition, serologic investigations in populations at risk in northern Italy have shown only a low prevalence of specific antibodies (0.6%–5%) (*2**,**3*). A surveillance system for TBEV infections was started after autochthonous TBEV was recognized in late summer and fall 2003 in Friuli-Venezia Giulia (FVG), a small region of northeastern Italy with nearly 1 million inhabitants (*4*). Surveillance is based on systematic microbiologic screening of all patients referred to the emergency departments of regional hospitals for suspected community-acquired central nervous system infections or fever and headache with a history of tick bite in the past 6 weeks. Screening for TBEV was performed on sera or cerebrospinal fluid (CSF) by enzyme immunoassay (Enzygnost Anti-TBE virus Ig, Dade Behring Marburg GmbH, Marburg, Germany) and repeated on convalescent-phase sera. Demonstration of specific immunoglobulin M (IgM) in serum or CSF in the acute phase or >4-fold rise in serum antibody titer in the convalescent phase was interpreted as an indicator of recent TBEV infection. For surveillance purposes, TBEV infection was defined when hemagglutination-inhibition antibody test and neutralization assay by a reference laboratory confirmed ELISA results (*5*). Data were collected at a regional reference center, where cases were classified as possible, probable, and confirmed, according to the new TBEV case definition (*6*).

From July 2003 through November 2005, 20 cases of TBEV infection were detected; their demographic, epidemiologic, and clinical characteristics are given in the Table. Cases occurred throughout the year, with a biphasic peak in June and September–November. A biphasic clinical course was reported in 10 patients. The median period between tick bite and date of referral to hospital was 22 days (range 15–46 days). Seventeen cases were classified as confirmed, 2 as probable, and 1 case could not be classified because symptoms started after tick season (December) (*6*). Two patients were coinfected with *Borrelia burgdorferi*.

**Table:** Demographic, epidemiologic, and clinical data of 20 patients with TBEV infection in Friuli-Venezia Giulia*

Patient	Sex	Age (y)	Tick bite	Hospitalization date (length of hospitalization [d])	Definitive diagnosis	Sequelae
1	F	36	Yes	2003 Jul 28 (31)	MEM	UL paresis
2	M	58	Yes	2003 Oct 13 (15)	E	Absent
3	F	42	Yes	2003 Oct 17 (19)	ME	Absent
4	F	27	No	2003 Dec 30 (25)	ME	UL paresis, paresis of VII cranial nerve
5	M	16	Yes	2004 Apr 28 (21)	ME	UL tremors
6	F	53	Yes	2004 Jun 21 (18)	ME	Diplopia
7	M	43	Yes	2004 Jul 17 (0)	FF	Absent
8	M	62	Yes	2004 Oct 10 (10)	ME	UL paresthesia
9	M	35	Yes	2004 Nov 8 (15)	ME	Absent
10	F	77	Yes	2004 Nov 22 (0)	FF	Absent
11	F	36	Yes	2005 May 8 (19)	MEM	UL paresis
12	M	12	Yes	2005 May 13 (27)	ME	Absent
13	M	64	Yes	2005 Jun 10 (11)	FF	UL paresthesia, hearing impairment
14	M	59	Yes	2005 Jun 20 (12)	M	Absent
15	M	15	Yes	2005 Sep 1 (10)	ME	Absent
16	F	39	Yes	2005 Sep 8 (8)	M	Absent
17	M	70	Yes	2005 Sep 16 (53)	MEM	UL paresis, RI, VAP
18	M	75	No	2005 Oct 18 (10)	FF	UL tremors
19	M	20	No	2005 Nov 2 (7)	M	UL tremors
20	M	61	Yes	2005 Nov 26 (13)	E	UL tremors, ataxia, opsoclonus

*TBEV, tickborne encephalitis virus; MEM, meningoencephalomyelitis; UL, upper limbs; E, encephalitis; ME, meningoencephalitis; FF, febrile form; M, meningitis; RI, respiratory insufficiency; VAP, ventilator-associated pneumonia.

The most common symptoms were fever, headache, nausea, vomiting, and myalgia; the most common central nervous system signs were stiff neck, irritability, and limb paresis. Five patients only reported headache and fever without neurologic signs. Lumbar puncture, performed in 15 patients, showed mild pleocytosis with neutrophil predominance in 13 patients, elevated protein level in 14 patients, and normal glucose level in all.

The clinical syndrome was classified, in accordance with Kaiser et al., into febrile form (4 cases), aseptic meningitis (3 cases), encephalitis (2 cases), meningoencephalitis (8 cases), and meningoencephalomyelitis (3 cases) (*7*). None of the patients died, but 3 required respiratory support in the intensive care unit. Outcome was favorable for 9 patients; major neurologic sequelae were observed in 6 and minor sequelae in 5.

During the past 20 years, TBEV has reemerged in several European areas that had been disease free (*1**,**8*). In FVG, which borders disease-endemic areas such as Slovenia and Austria, the first cases of TBEV infection were documented recently (*4*). Several explanations, in addition to the well-established role of climate change, can be proposed (*1*). First, in Slovenia, after the end of the Communist regime, recreational activities increased considerably, with the creation of natural parks and hunting grounds, densely populated with deer, chamois, rodents, foxes, and other wild animals that can easily cross national borders (*9*). Second, after the 1976 earthquake that destroyed a large number of mountain villages in FVG, economic activities were progressively concentrated in the plains of the region, which rapidly increased urbanization of the plains towns. As a consequence, the mountains in the northern part of the region were progressively abandoned by humans and returned to wilderness. A final possible explanation is that TBEV cases were undiagnosed because awareness among local physicians was low; however, this variable likely played a minor role, since a recent serologic survey of persons at high risk (forest rangers) yielded a low positivity ratio (*3*). If even workers at risk had a low seroprevalence, TBEV cases were likely uncommon in the region.

The implementation of a regional active surveillance system allows the highest sensitivity in assessing the epidemiologic features of TBEV infections, which are characterized by highly disease-endemic microfoci in areas free of the problem (*10*). Precisely defining areas where risk is particularly will lead to optimal use of prevention programs and design of educational programs for residents, tourists, and healthcare workers.

## References

[1] Günther G, Haglund M. Tick-borne encephalopathies: epidemiology, diagnosis, treatment and prevention. CNS Drugs. 2005;19:1009–32. 16332143 10.2165/00023210-200519120-00004

[2] Cristofolini A, Bassetti D, Schallenberg G. Zoonoses transmitted by ticks in forest workers (tick-borne encephalitis and Lyme borreliosis): preliminary results. Med Lav. 1993;84:394–402. 8114653

[3] Cinco M, Barbone F, Ciufolini M, Mascioli M, Anguero Rosenfeld M, Stefanel P, Seroprevalence of tick-borne infections in forestry rangers from northeastern Italy. Clin Microbiol Infect. 2004;10:1056–61. 10.1111/j.1469-0691.2004.01026.x 15606631

[4] Beltrame A, Cruciatti B, Ruscio M, Scudeller L, Cristini F, Rorato G, Tick-borne encephalitis in Friuli Venezia Giulia, northeastern Italy. Infection. 2005;33:158–9. 10.1007/s15010-005-4109-1 15940419

[5] Holzmann H, Kundi M, Stiasny K, Clement J, McKenna P, Kunz C, Correlation between ELISA, hemagglutination inhibition, and neutralization tests after vaccination against tick-borne encephalitis. J Med Virol. 1996;48:102–7. 10.1002/(SICI)1096-9071(199601)48:1<102::AID-JMV16>3.0.CO;2-I 8825718

[6] Stefanoff P, Eidson M, Morse DL, Zielinski A. Evaluation of tickborne encephalitis case classification in Poland. Euro Surveill. 2005;10:23–5. 15701936

[7] Kaiser R. The clinical and epidemiological profile of tick-borne encephalitis in southern Germany 1994–98: a prospective study of 656 patients. Brain. 1999;122:2067–78. 10.1093/brain/122.11.2067 10545392

[8] Skarpaas T, Ljostad U, Sundoy A. First human cases of tickborne encephalitis, Norway. Emerg Infect Dis. 2004;10:2241–3. 15663873 10.3201/eid1012.040598 PMC3323397

[9] Lesnicar G, Poljak M, Seme K, Lesnicar J. Pediatric tick-borne encephalitis in 371 cases from an endemic region in Slovenia, 1959 to 2000. Pediatr Infect Dis J. 2003;22:612–7. 10.1097/00006454-200307000-00009 12867836

[10] Zeman P. Objective assessment of risk maps of tick-borne encephalitis and Lyme borreliosis based on spatial patterns of located cases. Int J Epidemiol. 1997;26:1121–9. 10.1093/ije/26.5.1121 9363536

